# Continuous Monitoring of Positive Airway Pressure Therapy with a Smartphone-Based Home Sleep Apnea Test

**DOI:** 10.3390/medicina62061008

**Published:** 2026-05-22

**Authors:** Sungjin Heo, Seunghun Kim, Sungeun Moon, Sujin Lee, Dongheon Lee, Joonki Hong, Yoo-Sam Chung, Hyun Jik Kim, Jung Kyung Hong, In-Young Yoon, Jeong-Whun Kim

**Affiliations:** 1 Asleep Research Institute, Seoul 06159, Republic of Korea; chris.heo@asleep.ai (S.H.); snorlax@asleep.ai (S.K.); tessa.moon@asleep.ai (S.M.); sue.lee@asleep.ai (S.L.); sleep@asleep.ai (D.L.); nocturne@asleep.ai (J.H.); 2 Department of Otorhinolaryngology-Head and Neck Surgery, Asan Medical Center, College of Medicine, University of Ulsan, Seoul 05505, Republic of Korea; yschung@amc.seoul.kr; 3 Department of Otorhinolaryngology, Seoul National University College of Medicine, Seoul National University Hospital, Seoul 03080, Republic of Korea; hyunjerry@snu.ac.kr; 4 Department of Psychiatry, Seoul National University College of Medicine, Seoul National University Bundang Hospital, Seongnam 13620, Republic of Korea; 5 Department of Otorhinolaryngology-Head and Neck Surgery, Seoul National University College of Medicine, Seoul National University Bundang Hospital, 82 Goomiro 173-Beongil, Bundang-gu, Seongnam 13620, Republic of Korea

**Keywords:** obstructive sleep apnea, positive airway pressure, home sleep apnea test, artificial intelligence

## Abstract

*Background and Objectives*: Adherence to positive airway pressure (PAP) is often suboptimal, and current monitoring relies on device logs that, by design, cannot detect respiratory events outside the therapy window. This creates a physiological blind spot during periods of non-usage. This study aimed to demonstrate the clinical necessity of independent, continuous monitoring using a smartphone-based home sleep apnea test (S-HSAT) by validating treatment effectiveness on adherent nights and quantifying the untreated apnea burden caused by partial adherence. *Methods*: We prospectively monitored 63 obstructive sleep apnea (OSA) patients commencing PAP therapy. Nightly apnea–hypopnea index (AHI) and usage time were recorded simultaneously by an S-HSAT (ApnoTrack) and the PAP device over a 30-day period. Nights were categorized by the duration discrepancy between S-HSAT and PAP (full-use, ≤5 min; intermediate-use, 5–30 min; partial-use, >30 min) using physiologically and operationally derived thresholds. *Results*: Final analysis included 39 participants contributing 667 nights (24 participants excluded due to non-use of one or both devices). Full-use nights (46.2%) showed close agreement between S-HSAT and PAP mean AHI (2.8 ± 4.3 vs. 2.5 ± 2.0 events/h; *p* = 0.13). On intermediate-use and partial-use nights (20.7% and 33.1%, respectively), substantial AHI discrepancies emerged (7.3 ± 5.5 vs. 3.8 ± 3.3 and 11.0 ± 7.4 vs. 2.8 ± 2.5 events/h, respectively; both *p* < 0.001). *Conclusions*: Independent S-HSAT monitoring quantified an untreated apnea burden that is invisible to PAP logs alone, while confirming therapeutic efficacy on well-adherent nights. These findings suggest that continuous independent monitoring may help bridge the gap between prescribed therapy and actual physiological outcomes in OSA care.

## 1. Introduction

Obstructive sleep apnea (OSA) is a common sleep disorder characterized by repeated upper-airway collapse during sleep [1]. Patients with untreated severe OSA face markedly higher risks of cardiovascular events [2], which are reduced with effective treatment [3]. Positive airway pressure (PAP) remains the gold-standard therapy, preventing airway collapse via pressurized airflow [4,5]. It is typically indicated for patients with moderate to severe OSA (apnea–hypopnea index (AHI) ≥ 15), or mild OSA (AHI ≥ 5) accompanied by subjective sleepiness [6].

Despite its efficacy, PAP adherence remains a major challenge. Mask discomfort, device noise, nasal symptoms, and other side effects reduce long-term acceptability, and non-adherence rates have consistently been reported at approximately 30–40% [7]. Because adequate nightly usage (typically ≥4 h) is essential for clinical benefit, whether prescribed therapy translates into actual physiologic disease control during real-world use becomes a central clinical question, one that motivates continuous, objective monitoring of both adherence and treatment effectiveness rather than reliance on intermittent follow-up alone [8]. The need is further amplified by the substantial night-to-night variability in apnea severity, influenced by factors such as sleep stages, body position, and nasal congestion [9]; standard follow-up based on intermittent device downloads or patient reports often fails to capture this variability, and remote-monitoring strategies have therefore been proposed to improve adherence [10].

Critically, PAP device logs reflect only the periods when the device is in use; respiratory events occurring after early mask removal or during intra-night interruptions are systematically invisible to current monitoring. An independent, contactless monitor that captures the entire sleep period is therefore needed to bridge this physiological blind spot. Among candidate modalities, a smartphone-based monitor is uniquely attractive because it leverages a device already present in the patient’s bedroom, requires no consumables or additional hardware, and is suitable for daily, long-term use. Recent deep learning advancements have enabled contactless, sound-based AI for sleep analysis: such systems analyze nocturnal breathing sounds to detect apneas, hypopneas, and sleep stages without requiring physical sensors, and prior studies have validated that such acoustic analysis can accurately estimate OSA severity compared with standard polysomnography (PSG) [11,12,13].

Existing alternatives each illustrate the limitations that a smartphone-based home sleep apnea test (S-HSAT) can address. Telemonitoring of PAP-recorded metrics, while widely available, cannot capture events outside the therapy window; wrist-worn HSATs and under-mattress sensors extend coverage but are limited by consumable costs, contact-related discomfort, or the need to install dedicated hardware. Smartphone-based, sound-only monitoring addresses these limitations simultaneously, offering a low-burden modality that may be deployed at scale. To our knowledge, this is the first study to deploy an S-HSAT in parallel with PAP therapy in order to quantify the burden of untreated apnea during periods of non-use. We hypothesized that reliance on PAP data alone may create a “blind spot” during non-adherence; by validating S-HSAT accuracy on adherent nights and, crucially, quantifying untreated apnea during partial adherence, we aimed to demonstrate how independent monitoring can bridge the gap between prescribed therapy and actual physiological outcomes.

## 2. Materials and Methods

### 2.1. Study Design and Participants

We conducted a single-center, prospective observational study involving adults newly prescribed PAP therapy for OSA. Participants were recruited from the sleep clinic at Seoul National University Bundang Hospital between April 2025 and February 2026, and the study protocol was approved by the Institutional Review Board (IRB No. B-2504-965-303). Written informed consent was obtained from all subjects. Inclusion criteria were age ≥ 19 years and an OSA diagnosis confirmed by standard PSG. All eligible patients were newly initiated on PAP and instructed to use the device throughout the 30-day monitoring period. Patients who were not candidates for PAP or declined PAP therapy were excluded. Pre-specified non-use of either device, 4 participants who did not initiate PAP and 20 who did not use the S-HSAT application, was treated as a data-quality exclusion prior to analysis, rather than as outcome-based filtering.

### 2.2. S-HSAT and PAP Data Collection

**Figure 1:**
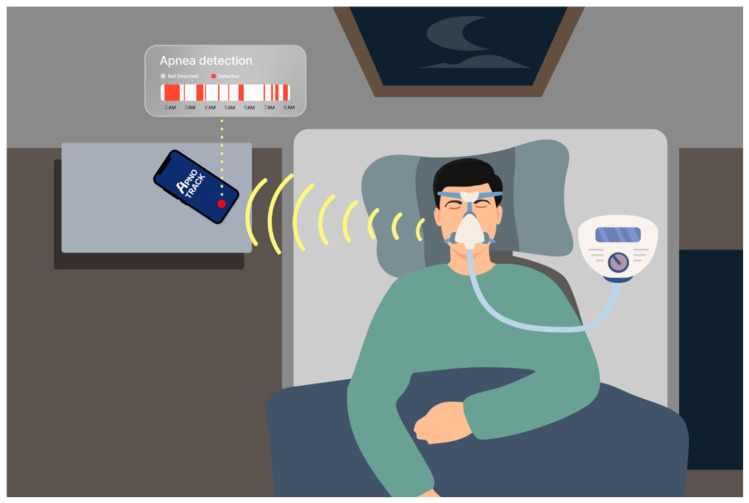
Overview of the experimental setup. The patient undergoes positive airway pressure (PAP) therapy while simultaneously utilizing the smartphone-based home sleep apnea test (S-HSAT) application placed at the bedside. Both systems independently record the Apnea–Hypopnea Index (AHI) and usage duration throughout the night, allowing for comparative data analysis.

To monitor sleep concurrently with PAP therapy, participants utilized ApnoTrack (Asleep Inc., Seoul, Republic of Korea), an S-HSAT application built on a sound-based AI. Building on prior evidence that smartphone-recorded breathing sounds can be used to estimate OSA severity [11], the AI predicting OSA events was first developed using home noises and PSG audio [12] and subsequently validated on smartphone-recorded data against home polysomnography in adult OSA cohorts [13]. In this in-home validation, at an AHI threshold of 15 events/h, the AI demonstrated sensitivity and specificity of 90.9% and 94.4% on Android smartphones and 90.0% and 94.4% on iOS, respectively. On the basis of these studies, the application received Class II medical-device approval from the Korean Ministry of Food and Drug Safety (MFDS) and is currently used in routine clinical practice in Korea. Acknowledged technical limitations include sensitivity to ambient noise, dependence on smartphone microphone characteristics, the absence of oxygen-saturation data, and the assumption of a single primary breathing source within the recording field. Participants were guided by the standard ApnoTrack user manual, which recommends single-occupancy use, smartphone placement within approximately 1 m of the head of the bed, and at least 4 h of recording per night, with the application activated before sleep onset (Figure 1). The application provided nightly estimates of AHI and sleep metrics, such as total sleep time, time in bed, and sleep stages. In this analysis, ‘time in bed’ was used to represent the monitoring duration derived from the S-HSAT.

Concurrently, all participants were treated using a PAP device (AirSense 10 AutoSet; ResMed, San Diego, CA, USA) configured in auto-titrating mode with a pressure range of 5–12 cmH_2_O. Because the device adjusts pressure dynamically each night to suppress respiratory events, a separate in-laboratory titration session was not performed prior to home initiation, in keeping with current institutional practice for auto-PAP therapy. PAP machine data, including device-derived AHI and nightly usage duration, were retrieved via memory cards or the manufacturer-specific cloud platform. Participants were instructed to use their PAP device as prescribed while simultaneously monitoring their sleep using the S-HSAT application throughout the study period. To assess baseline therapeutic compliance, we evaluated overall patient adherence using the conventional clinical definition of average nightly PAP usage ≥ 4 h.

### 2.3. Classification of PAP Adherence Levels

Each recorded night was categorized into one of three adherence groups based on the temporal discrepancy between the S-HSAT-derived usage time ($T_{HSAT}$) and the PAP usage time ($T_{PAP}$). The time difference ($∆T=\;T_{HSAT}-\;T_{PAP}$) was used to classify nights as follows:

- Full-use nights (∆T ≤ 5 min): Nights where the PAP usage duration matched or exceeded the S-HSAT usage time, allowing for a negligible margin of ≤5 min. This indicates comprehensive therapeutic coverage, implying that PAP was used continuously throughout the entire sleep period.
- Intermediate-use nights (5 < ∆T ≤ 30 min): Nights in which PAP was worn for most, but not all, of the sleep period, resulting in a time difference of more than 5 min but no more than 30 min. This reflects brief lapses in PAP use during sleep.
- Partial-use nights (∆T > 30 min): Nights where PAP usage was shorter than the S-HSAT usage time by more than 30 min, indicating substantial periods of untreated sleep, typically due to early mask removal.

The 5 min lower boundary reflects the typical usage pattern in our cohort, in which participants generally removed the PAP mask upon awakening (ending PAP) and subsequently stopped the S-HSAT application. The 30 min threshold was chosen to distinguish brief, transient interruptions (e.g., physiological needs or device adjustments) from clinically meaningful periods of untreated sleep; because apnea events and oxygen desaturation are known to recur rapidly after PAP discontinuation [14], interruptions exceeding 30 min are likely to represent a substantial exposure to untreated OSA. Alternative threshold configurations were also examined, and the resulting AHI patterns were broadly consistent with the chosen scheme.

### 2.4. Data Analysis

Comparative analyses between S-HSAT and PAP measurements were performed across the three adherence categories. For each category, descriptive statistics were calculated as mean ± standard deviation (SD) for AHI and usage duration. Paired *t*-tests compared S-HSAT and PAP-derived AHI within each adherence category; mean differences are reported with 95% confidence intervals (CIs) and Cohen’s d effect sizes for paired data.

To assess reliability and agreement between S-HSAT and PAP-derived AHI, we calculated the Concordance Correlation Coefficient (CCC) and Intraclass Correlation Coefficient (ICC(2,1)). Bland–Altman analyses were performed to assess bias and 95% limits of agreement (LoA). Throughout the analysis, PAP-recorded AHI was treated as a clinical comparator, not as a diagnostic gold standard.

No quality-based filtering was applied; all nights with concurrent valid measurements from both devices were included in the analysis. Nights for which either device did not produce a recording could not be paired and were therefore not used in the comparisons. A *p*-value < 0.05 was considered statistically significant. All analyses were conducted using SPSS version 25.0 for Windows (SPSS Inc., Chicago, IL, USA).

## 3. Results

Throughout this section, PAP-recorded AHI refers to residual respiratory events detected by the device while therapy was being delivered, whereas S-HSAT-derived AHI reflects respiratory events across the entire sleep period, including any time when the patient was not on PAP. The two metrics are therefore not interchangeable, and any divergence between them quantifies physiologic disease activity that occurred outside the therapy window.

### 3.1. Participant Characteristics

Of the initial 63 enrolled participants, 39 participants who completed the monitoring were included in the final analysis. The mean age of the study population was 45.1 ± 11.1 years. The average body mass index (BMI) was 26.4 ± 5.2 kg/m^2^, categorizing the study cohort as obese according to the Asia-Pacific obesity criteria (BMI ≥ 25 kg/m^2^) [15]. Diagnostic PSG revealed a mean AHI of 34.0 ± 18.6 events/h, indicating predominantly severe OSA. Subjective sleep was poor (Pittsburgh Sleep Quality Index 7.6 ± 3.2), and daytime sleepiness was clinically relevant (Epworth Sleepiness Scale 8.9 ± 5.2). Baseline characteristics are summarized in Table 1.

### 3.2. PAP Usage Patterns

**Figure 2:**
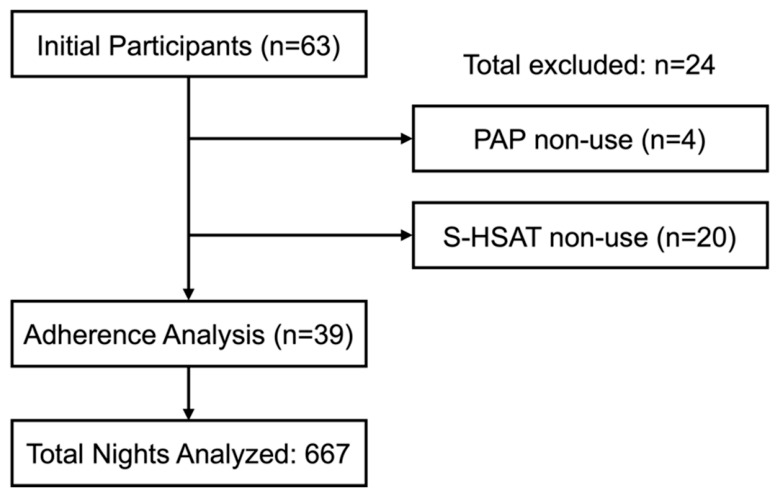
Study flow diagram of participant selection and exclusion. The diagram details the study enrollment and exclusion process. Of the 63 initial participants, 24 were excluded: 4 due to positive airway pressure (PAP) non-use and 20 due to smartphone-based home sleep apnea test (S-HSAT) non-use. Consequently, 39 participants were included in the final adherence analysis, yielding a total dataset of 667 monitored nights.

Twenty-four of the 63 participants were excluded prior to analysis because of pre-specified non-use of one or both devices: 4 did not initiate PAP therapy and 20 did not use the S-HSAT device. Comparison of baseline demographic and clinical characteristics between the 39 included and 24 excluded participants showed no statistically significant differences across age, body mass index, baseline polysomnographic AHI, Pittsburgh Sleep Quality Index, and Epworth Sleepiness Scale (Table 2). The remaining 39 patients (61.9%) contributed 667 nights of simultaneous monitoring (Figure 2). Of these, 308 (46.2%) were classified as full-use, 138 (20.7%) as intermediate-use, and 221 (33.1%) as partial-use. Full numerical comparisons of monitoring and usage duration by category are provided in Table 3; in brief, PAP usage exceeded S-HSAT duration by approximately 22 min on full-use nights (343.8 ± 70.0 vs. 321.7 ± 69.9 min; *p* < 0.001), whereas S-HSAT duration exceeded PAP usage on intermediate-use nights (333.8 ± 80.3 vs. 313.4 ± 80.8 min; *p* < 0.001) and substantially so on partial-use nights (381.7 ± 93.5 vs. 259.8 ± 92.6 min; *p* < 0.001).

### 3.3. AHI by Adherence Category

**Figure 3:**
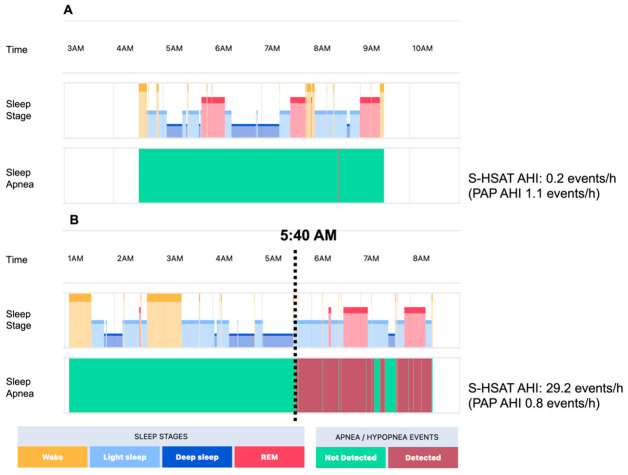
Representative examples of apnea event detection visualized on the clinical dashboard. The tracks display apnea/hypopnea events detected by the smartphone-based home sleep apnea test (S-HSAT) alongside sleep stages over time. (**A**) A full-use night exhibiting high concordance between devices. Sleep apnea was well-controlled, with S-HSAT estimating an AHI of 0.2 events/h and the positive airway pressure (PAP) device reporting an AHI of 1.1 events/h. (**B**) A partial-use night from the same patient. Early PAP removal (around 5:00 a.m.) resulted in a surge of apnea events detected by S-HSAT but missed by the PAP device. Consequently, S-HSAT estimated an AHI of 29.2 events/h, in contrast to the PAP-reported AHI of 0.8 events/h.

On full-use nights, S-HSAT-derived AHI did not differ from PAP-derived AHI (2.8 ± 4.3 vs. 2.5 ± 2.0 events/h; mean difference 0.31 events/h, 95% CI −0.13 to 0.75; Cohen’s d = 0.09; *p* = 0.13). Figure 3A presents a representative full-use night from a patient with severe baseline OSA (PSG AHI 88.8 events/h) showing concordant low estimates from both devices (S-HSAT 0.2 vs. PAP 1.1 events/h).

In contrast, significant discrepancies emerged on nights with incomplete adherence. On intermediate-use nights, S-HSAT AHI exceeded PAP AHI (7.3 ± 5.5 vs. 3.8 ± 3.3 events/h; mean difference 3.50 events/h, 95% CI 2.84 to 4.16; Cohen’s d = 0.85; *p* < 0.001). This divergence was largest on partial-use nights, where S-HSAT AHI reached 11.0 ± 7.4 events/h compared with a PAP-derived AHI of 2.8 ± 2.5 events/h (mean difference 8.29 events/h, 95% CI 7.40 to 9.18; Cohen’s d = 1.19; *p* < 0.001). Clinically, the partial-use S-HSAT mean AHI falls within the moderate-OSA range, whereas the corresponding PAP-AHI would be classified as well-controlled, illustrating the magnitude of the gap. Figure 3B shows a partial-use night from the same participant as Figure 3A: following PAP termination at approximately 05:00, S-HSAT detected frequent apneas during the unmonitored period, yielding a nightly S-HSAT AHI of 29.2 events/h while the PAP-recorded AHI remained at 0.8 events/h.

### 3.4. Agreement and Concordance

**Figure 4:**
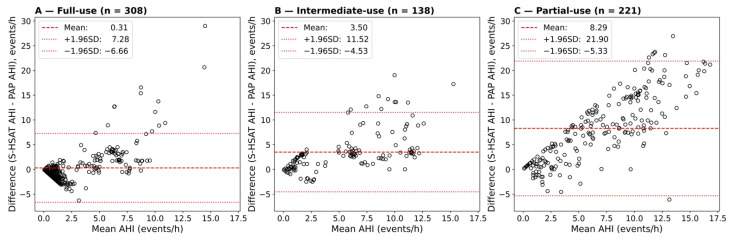
Night-level Bland–Altman plots of agreement between smartphone-based home sleep apnea test (S-HSAT) and positive airway pressure (PAP) AHI measurements. The dashed red lines represent the mean difference (bias), and the dotted red lines indicate the 95% limits of agreement (LoA). (**A**) Full-use nights (n = 308) exhibit minimal mean bias (+0.31 events/h; 95% LoA: −6.66 to 7.28), indicating high concordance. (**B**) Intermediate-use nights (n = 138) show increased mean bias (+3.50 events/h; 95% LoA: −4.53 to 11.52). (**C**) Partial-use nights (n = 221) demonstrate substantial positive bias (+8.29 events/h; 95% LoA: −5.33 to 21.90).

Agreement between S-HSAT and PAP-derived AHI was evaluated by adherence category (Table 4). On full-use nights, CCC and ICC were 0.43 and 0.43, respectively, with a Bland–Altman mean bias of 0.31 events/h (95% LoA −6.66 to 7.28; Figure 4A). On intermediate-use nights, CCC and ICC were 0.46 and 0.46, with a mean bias of 3.50 events/h (95% LoA −4.53 to 11.52; Figure 4B). Concordance was lowest on partial-use nights, CCC and ICC were 0.10 and 0.10, with a mean bias of 8.29 events/h and widened limits of agreement (−5.33 to 21.90; Figure 4C).

## 4. Discussion

Our study demonstrates the clinical value of continuous at-home monitoring for OSA patients on PAP therapy through an independent S-HSAT. Independent nightly monitoring identified residual sleep-disordered breathing that may be missed when relying only on PAP device-reported AHI; S-HSAT tracked increases in estimated AHI after early PAP discontinuation while providing broadly similar AHI estimates to PAP on fully adherent nights. These findings have important implications for patient management, underscoring the need for independent, daily monitoring to verify therapeutic effectiveness and uncover therapeutic gaps masked by device-generated reports.

### 4.1. PAP and S-HSAT Usage Patterns

Distinct operational characteristics produced systematic differences between the two devices. On full-use nights, PAP usage exceeded the S-HSAT monitoring duration by approximately 22 min (343.8 vs. 321.7 min). This is consistent with the typical operational sequence: participants generally activate the PAP device prior to lying down, whereas the S-HSAT application is initialized only after settling into bed. Conversely, upon awakening, the application was terminated immediately while PAP devices may continue to log usage owing to residual airflow before mask removal. Consequently, PAP usage logs could tend to slightly overestimate the actual sleep period covered by therapy.

On intermediate-use and partial-use nights, the time differences were approximately 20 and 122 min, respectively. Given the systematic 22 min over-logging observed on full-use nights, the PAP logs likely overestimate effective therapy time on these nights as well; the actual period of untreated sleep is therefore likely longer than the nominal discrepancies suggest.

### 4.2. Validation of AHI Estimation Under Full PAP Adherence

On full-use nights, S-HSAT and PAP produced similar mean AHI estimates, suggesting that S-HSAT may provide complementary monitoring information when PAP use is continuous. Establishing alignment with the current clinical comparator for home monitoring is a fundamental step, supporting the use of S-HSAT as a viable adjunct for long-term follow-up strategies. We note that the underlying AI has previously been validated against home polysomnography in adult OSA cohorts using actual smartphone-recorded data [13], building on earlier development against home noises and PSG audio [12] and on prior evidence of feasibility for sound-based OSA prediction [11]; on the basis of these validation studies, ApnoTrack received Class II medical-device approval from the Korean Ministry of Food and Drug Safety (MFDS) and is currently used in routine clinical practice in Korea. The present study therefore complements, rather than replaces, those diagnostic validations by demonstrating longitudinal clinical utility under real-world PAP therapy.

### 4.3. Quantification of Untreated Apnea Burden During Partial Adherence

A distinct capability of independent S-HSAT is continuous coverage of the entire sleep period, beyond the therapeutic window captured by PAP devices. This capability proved crucial in our cohort, where patients discontinued therapy before sleep ended on nearly half of the recorded nights (combining intermediate-use and partial-use). PAP logs indicated effective control during active use, while S-HSAT analysis captured a substantial return of disease activity following device removal. Specifically, even on partial-use nights where average PAP usage exceeded the conventional 4 h adherence threshold, the mean S-HSAT AHI rebounded to the moderate range (11.0 events/h), a finding that diverges sharply from the lower metrics reported by the PAP device. Reliance solely on PAP-derived data may therefore underestimate the true nightly disease burden, potentially obscuring residual risks such as oxygen desaturation, sleep fragmentation, and the cumulative cardiovascular and cognitive sequelae of untreated OSA documented in long-term cohorts [2,3].

### 4.4. Clinical Importance of Monitoring and Compliance

Full PAP adherence was achieved on only 46.2% of monitored nights. This is consistent with well-known challenges in real-world practice, where suboptimal adherence may prevent patients from realizing the full dose-dependent benefits of therapy, such as blood pressure reduction and improved daytime alertness [16,17]. Because PAP logs cannot assess disease burden during non-usage, relying solely on them leaves a critical blind spot in patient management. Monitoring compliance is therefore insufficient by itself; independent verification of actual physiological control is essential to ensure therapeutic success.

Translating these adherence categories into bedside use, full-use nights provide reassurance that prescribed therapy is delivering physiological control and may be used to support continued therapy. Intermediate-use nights, characterized by brief lapses, identify patients who are essentially adherent but who may benefit from targeted interventions such as mask-fit optimization or sleep-hygiene counseling. Partial-use nights are clinically the most consequential: they correspond to patients whose mean nightly use may exceed the conventional 4 h adherence threshold yet who experience long, untreated periods of disease activity invisible to PAP logs. Identifying this subgroup may allow clinicians to intervene before the cumulative cardiovascular and cognitive risks of intermittent untreated OSA accrue.

Prior studies have attempted to bridge this gap using independent modalities. A study using Watch PAT, a wrist-worn HSAT device, monitored patients on PAP and identified that nearly half of those with clinically suspected residual sleep-disordered breathing despite ongoing CPAP had elevated residual AHI levels [18]. Studies using contactless under-mattress sensors have reported significant discrepancies, with independent monitoring detecting a mean AHI of 10.9 events/h compared with 1.9 events/h from PAP logs [19]. Despite their value, widespread adoption of these approaches has been limited by the cost of disposable HSAT consumables or the logistical burden of installing additional hardware. Contactless, sound-based AI may offer a complementary path forward by leveraging a device already present in the patient’s environment.

### 4.5. Implications for Long-Term Management, Behavior, and Device Optimization

Beyond identifying gaps, S-HSAT monitoring may shape three complementary aspects of long-term care. First, by lowering the cost and burden of independent measurement, it allows physiologic outcomes to be tracked across months rather than at single follow-up visits. Second, providing patients with daily, individualized feedback that visualizes the immediate return of respiratory events after early mask removal can serve as a behavioral lever, complementing existing telemedicine-based education programs. Third, persistent S-HSAT-detected events during reported PAP use can prompt timely device-side optimization (for example, pressure adjustment, mask refitting, or escalation to bilevel therapy) before patients accrue the chronic consequences of incompletely treated OSA.

### 4.6. Practical Barriers

Several practical barriers must be addressed before broad implementation. Patient adherence to nightly application activation is itself non-trivial; in our cohort, 20 of 63 participants did not use the application sufficiently for inclusion, indicating that engagement strategies will be required for less digitally fluent populations. Formal cost-effectiveness analyses comparing S-HSAT-augmented care with standard PAP follow-up alone have not yet been conducted; quantifying whether the added clinical value (e.g., earlier identification of partial adherence, behavioral feedback, more responsive therapy adjustment) justifies the additional implementation cost is an important next step. Additional validation work specific to the PAP-monitoring context will also strengthen interpretation. While the underlying AI has been validated against home polysomnography in adult OSA cohorts, validation against concurrent PSG specifically during active PAP therapy has not yet been performed and would provide a more direct gold-standard reference than PAP-derived AHI. The S-HSAT also does not capture oxygen saturation, and comparing S-HSAT-detected events with concurrent desaturation patterns would provide additional physiological grounding for the nightly AHI estimates obtained during PAP use.

### 4.7. Limitation

Our study has several limitations. First, the analysis was conducted with a relatively small cohort of 39 participants from a single center, and the population was homogeneous (predominantly Asian males with moderate-to-severe OSA). Additional research is needed to generalize these findings to females, other ethnic groups, patients with milder disease severity, or those treated with alternative modalities such as oral appliances.

Second, the validation in this study used PAP-derived AHI as a comparator rather than as a diagnostic gold standard. PAP event-detection algorithms are proprietary, manufacturer-specific, and based on airflow-only signal processing; they are not equivalent to PSG. Importantly, PAP-recorded AHI is by design restricted to periods of active therapy and therefore cannot, by construction, capture respiratory events occurring during non-usage. Our quantification of those events on intermediate- and partial-use nights should therefore be interpreted as an empirical demonstration of this gap rather than as a discrepancy attributable to S-HSAT measurement error.

Third, the primary objective of this study was to demonstrate the clinical utility and necessity of independent monitoring rather than to evaluate its impact on patient behavior. Whether S-HSAT-driven daily feedback improves long-term PAP adherence remains to be confirmed in future randomized trials.

Fourth, our analysis was subject to selection bias, as it inherently favored patients who were at least partially adherent to therapy and capable of operating the monitoring application. A substantial portion of the initial cohort (24 of 63) was excluded because of pre-specified non-use of PAP or the S-HSAT application. However, comparison of baseline demographic and clinical characteristics between included and excluded participants showed no statistically significant differences (Table 2). These results suggest that the analyzed cohort is broadly comparable to the originally enrolled population on measured baseline characteristics. Nevertheless, generalization to populations with greater demographic diversity, lower digital literacy, or markedly poorer adherence will require dedicated future studies.

Finally, the present analysis was conducted at the night level for paired device comparison and did not include a within-subject characterization of night-to-night variability. A rigorous treatment of that question requires individual-level matching of repeated measures across nights and a study design tailored to that aim, which is the focus of an ongoing follow-up study.

## 5. Conclusions

Our findings underscore the necessity of continuous independent monitoring for OSA patients on PAP. S-HSAT monitoring quantified a substantial untreated apnea burden on nights with poor adherence, a physiological gap that is invisible to PAP device data alone, while concurrently confirming therapeutic efficacy on well-adherent nights. This dual capability positions independent monitoring as a practical tool for both verifying therapeutic success and identifying adherence failures, and may bridge the gap between prescribed therapy and actual physiological outcomes. By providing detailed daily feedback, it may further empower patients and clinicians to address problems promptly, whether through improving mask comfort, adjusting therapy settings, or providing behavioral support, with the aim of maximizing the long-term benefits of treatment.

On the basis of these findings, we suggest two practical recommendations: continuous S-HSAT monitoring during the first 30 days of PAP initiation, when non-adherence risk is highest; and dedicated follow-up for patients in whom the S-HSAT/PAP AHI gap exceeds 3 events/h on at least 30% of nights. Patients most likely to benefit include those in the early adaptation phase of PAP, those with persistent symptoms despite reportedly adequate adherence, and those with moderate-to-severe baseline OSA in whom even brief untreated periods may carry meaningful risk. Given the single-center, demographically homogeneous nature of our cohort, larger multicenter studies, including women, additional ethnic groups, and patients across the full spectrum of OSA severity, are required before S-HSAT-augmented monitoring can be broadly recommended in routine clinical care.

## Figures and Tables

**Table 1:** Baseline characteristics of participants included in final analysis (39 patients, 667 nights).

Variable	Value
**Monitoring overview**	
Total analyzed nights	667
Nights per patient	17.1
**Demographics**	
Male sex, n (%)	39 (100)
Age, years	45.1 ± 11.1
Body mass index, kg/m^2^	26.4 ± 5.2
**Baseline OSA severity**	
Apnea–hypopnea index, events/h	34.0 ± 18.6
Severe OSA (≥30), n (%)	20 (51.3)
**Sleep-related questionnaires**	
Pittsburgh Sleep Quality Index	7.6 ± 3.2
Epworth Sleepiness Scale	8.9 ± 5.2

Data are presented as mean ± SD or number (%).

**Table 2:** Baseline characteristics of included vs. excluded participants.

Characteristic	Included (n = 39)	Excluded (n = 24)	*p* Value
Age, years	45.1 ± 11.1	46.5 ± 10.0	0.61
Sex, male n (%)	39 (100)	24 (100)	—
BMI, kg/m^2^	26.4 ± 5.2	27.6 ± 3.9	0.31
PSG AHI, events/h	34.0 ± 18.6	36.1 ± 24.1	0.72
Pittsburgh Sleep Quality Index	7.6 ± 3.2	8.5 ± 3.1	0.29
Epworth Sleepiness Scale	8.9 ± 5.2	11.1 ± 4.5	0.09

Data are presented as mean ± SD or n (%); *p* values from independent *t*-test for continuous variables. No measured variable differed significantly between groups (all *p* ≥ 0.09).

**Table 3:** Comparison of smartphone-based home sleep apnea test (S-HSAT) and positive airway pressure (PAP) measurements across adherence categories.

Measurement	Full-Use (308 Nights)	Intermediate-Use (138 Nights)	Partial-Use (221 Nights)
**AHI (events/h)**			
S-HSAT	2.81 ± 4.28	7.30 ± 5.53	11.04 ± 7.41
PAP	2.51 ± 2.01	3.80 ± 3.31	2.75 ± 2.48
*p*-value	0.13	<0.001	<0.001
*Cohen’s d*	0.09	0.85	1.19
**Usage Time (min)**			
S-HSAT	321.68 ± 69.86	333.81 ± 80.31	381.68 ± 93.54
PAP	343.82 ± 70.02	313.37 ± 80.82	259.77 ± 92.62
*p*-value	<0.001	<0.001	<0.001
*Cohen’s d*	−0.70	2.70	1.51

Data are presented as mean ± SD. Abbreviations: AHI, Apnea–Hypopnea Index; S-HSAT, Smartphone-based Home Sleep Apnea Test; PAP, Positive Airway Pressure.

**Table 4:** Night-level AHI Agreement Metrics Between S-HSAT and PAP Measurements across different adherence levels.

Metric	Full-Use	Intermediate-Use	Partial-Use
Number of nights	308	138	221
Concordance correlation coefficient	0.43	0.46	0.10
Intraclass correlation coefficient (two-way random effects, single measurement)	0.43	0.46	0.10
Mean bias, events per hour *	0.31	3.50	8.29
95% limits of agreement, events per hour	−6.66 to 7.28	−4.53 to 11.52	−5.33 to 21.90

* Mean bias calculated as the AHI estimated by the S-HSAT minus the AHI reported by the PAP device. All analyses were performed at the night level.

## Data Availability

The datasets generated and analyzed during the current study are not publicly available due to patient privacy and ethical restrictions regarding patient health information.
